# Interactions in the (Pre)metastatic Niche Support Metastasis Formation

**DOI:** 10.3389/fonc.2019.00219

**Published:** 2019-04-24

**Authors:** Ginevra Doglioni, Sweta Parik, Sarah-Maria Fendt

**Affiliations:** ^1^Laboratory of Cellular Metabolism and Metabolic Regulation, VIB-KU Leuven Center for Cancer Biology, VIB, Leuven, Belgium; ^2^Laboratory of Cellular Metabolism and Metabolic Regulation, Department of Oncology, KU Leuven and Leuven Cancer Institute, Leuven, Belgium

**Keywords:** metastatic niche, premetastatic niche, tumor environment, immune cells, stromal cells, extracellular matrix, nutrient environment, tumor secreted factors

## Abstract

Metastasis formation is the leading cause of death in cancer patients. Thus, understanding and targeting this process is an unmet need. Crucial steps during the establishment of metastases include the (pre)metastatic niche formation. This process relies on the interaction of the primary tumor with the environment of distant organs (premetastatic niche) and the interaction of cancer cells with their environment when arriving in a distant organ (metastatic niche). Here, we summarize the current knowledge on the interactions in the tumor environment that result in (pre)metastatic niche formation, specifically in the context of tumor secreted factors, extracellular matrix, immune as well as stromal cells, and nutrient availability. We further highlight strategies to disrupt these interactions as therapeutic interventions against metastases.

## Introduction

Cancer accounts for ~9.6 million deaths per year (1). The majority of these deaths are attributed to the formation of metastases, i.e., secondary tumors (2). Metastases are the final outcome of a cascade of events: first, cancer cells from a primary tumor invade the surrounding tissue and intravasate into the circulation. Subsequently, cancer cells seed and eventually colonize in a distant organ, which is a highly inefficient process since only 0.01% of cells that enter the circulation will succeed in colonizing a distant organ (3, 4). After colonizing a distant organ, cancer cells transition into a proliferative state which results in the establishment of metastases, i.e., secondary tumors. Importantly, the organ choice for metastasis formation is not random but at least in part directed by the primary tumor. For instance, colon cancer metastasizes mostly to the liver, breast cancer equally to the bone, liver, brain and lung, whereas prostate cancer most commonly results in bone metastases. While some organs such as liver, lung and bone are frequent metastatic sites, others, such as ovaries and skin are rarely home to secondary tumors (5). Strikingly, metastasizing cancer cells can be directed to different organs through tumor secreted factors (6). This finding proves the existence of a premetastatic niche, which creates a fertile environment for the seeding of disseminated cancer cells in selected secondary organs. Following seeding in a secondary organ, cancer cells interact dynamically with their environment, which creates the metastatic niche. These interactions include the cooperation with immune and stromal cells, the extracellular matrix and the organ nutrient environment (7, 8). Here, we highlight the interactions in (pre)metastatic niche and how they support metastasis formation.

### Formation of the Premetastatic Niche

Tumor cells require a permissive environment in terms of nutrients, extracellular matrix and immune cells to successfully seed in a distant organ. Nutrients are linked via the metabolic network to the ability of cancer cells to survive and seed in a certain environment (8). Consequently, increased availability of certain nutrients can support metastatic seeding and thus might be controlled in the premetastatic niche. The extracellular matrix constitutes a scaffold that supports the attachment and thus reactivation of survival signaling in cancer cells. Moreover, extracellular matrix components such as fibronectin and hyaluronan allow directed migration and enhancement of metabolic activity, respectively (9, 10). The structure of the organ intrinsic extracellular matrix is often less suited to support cancer cell attachment, metabolism and migration of recruited cells. Thus, remodeling of the extracellular matrix is an essential process in premetastatic niche formation. Finally, pro-tumor immune cells are enriched in the premetastatic niche to support cancer cell seeding via paracrine signaling and by suppressing anti-tumor immune cells. Some organ environments such as the lungs seem to have *per se* an environmental composition more supportive for cancer cells seeding (11–13). Additionally, primary tumors actively condition via secreted factors the nutrient, extracellular matrix and immune cell environment of a distant organ before the arrival of tumor cells and thus generate a permissive and supportive premetastatic niche (14).

Pro-tumor immune cells are important components of the premetastatic niche environment. Accordingly, it has been found that neutrophils are recruited to the lung premetastatic niche via factors secreted from the primary tumor (15–18). Specifically, it has been shown that neutrophils support the tumor initiating capacity of cancer cells that arrive in the premetastatic niche via leukotriene signaling (16) and that they create an immune suppressive environment by inhibiting anti-tumor CD8^+^ T cells (15). While patrolling monocytes can prevent successful seeding of cancer cells in the premetastatic niche (19, 20), macrophages, monocytes and bone marrow-derived cells are often part of a pro-tumor premetastatic niche (21–24). In this respect, it has been found that pancreatic cancer exosomes taken up by liver resident Kupffer cells induce, via TGFβ signaling, fibronectin production by hepatic stellate cells (23). This fibronectin enriched environment enhances recruitment of bone marrow-derived macrophages. Moreover, recent data suggest that tumor secreted factors modify perivascular cells to establish a pro-metastatic fibronectin-rich environment (25). These fibronectin enriched environments in turn recruit pro-tumor bone marrow-derived macrophages to the liver premetastatic niche (23). Beyond fibronectin additional extracellular matrix components are altered in the premetastatic niche to recruit pro-tumor stromal and immune cells. In line, activity of the enzyme lysyl oxidase (LOX) which crosslinks collagen of the extracellular matrix has been linked to the recruitment of myeloid cells to the lung premetastatic niche (26, 27). Accordingly, it has been found that primary breast cancers can secrete LOX (28). Extracellular matrix crosslinked through LOX activity is not only important for the recruitment of pro-tumor immune and stromal cells, but can also mediate osteoclast-driven premetastatic lesion formation in the bone (28, 29). This in turn enhances the chance that circulating tumor cells seed and colonize into bone metastases. Accordingly, it has been seen that HIF1α stabilization in osteoblast-lineage cells alters the bone extracellular matrix and thus dissemination of cancer cells to the bone (30). Additionally, cancer cells that seed in the premetastatic niche require certain nutrients. For instance it has been shown that breast cancer cells colonize the lung environment catabolize proline to sustain their energy needs (31) and rely on pyruvate to shape the metastatic niche environment (32). Moreover, metastasis initiating oral carcinoma depend on the fatty acid receptor CD36 (33). Thus, it is tempting to speculate that primary tumor secreted factors also create a permissive nutrient environment in the premetastatic niche. Accordingly, it has been observed that tumor secreted miRNA122 alters the metabolism of lung and brain resident cells to increase glucose availability in the premetastatic niche to boost the metabolism of arriving breast cancer cells (34).

**Figure 1:**
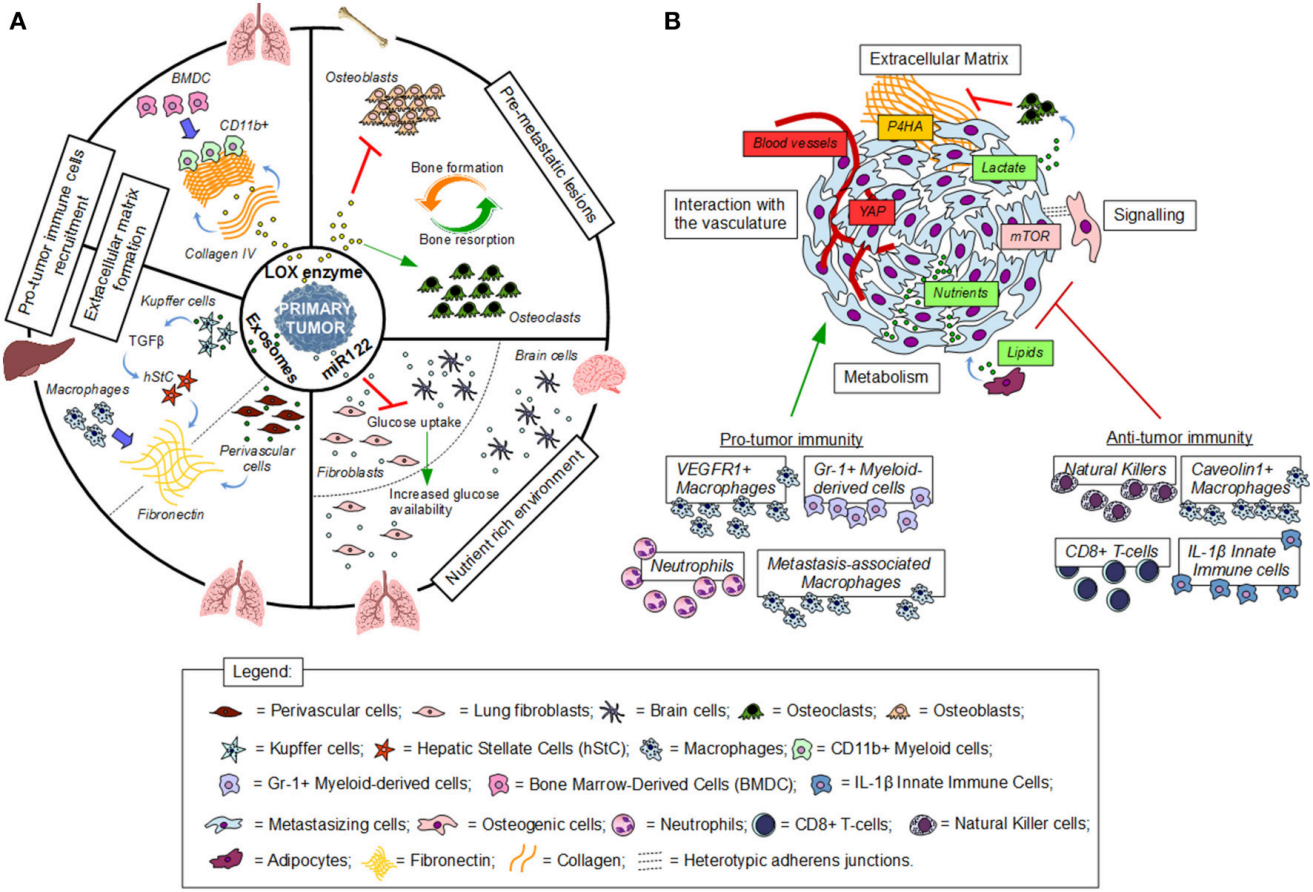
Establishment and interactions in the (pre)metastatic niche. **(A)** Premetastatic niche formation depends on the interaction of tumor secreted factors with the local environment of a distant organ. **(B)** Metastatic niche formation requires the interaction of cancer cells with the local environment. Red indicates inhibition, green indicates activation, blue thick arrows indicate recruitment.

Taken together, premetastatic niche formation is initiated by the primary tumor through secreted factors and boosts the chance that arriving cancer cells undergo successful seeding (Figure 1A).

### Interactions in the Metastatic Niche

After successful seeding in the premetastatic niche, cancer cells interact with their environment to promote their own metastatic outgrowth. These interactions are essential to evade immune destruction, activate growth signaling and gain access to nutrients that support their proliferation.

Immune cells such as natural killer cells, CD8^+^ T-cells, interleukin-1β-expressing innate immune cells and caveolin-1 expressing metastasis-associated macrophages have been implicated in preventing metastasis formation. Specifically, it has been observed that these cell types can kill metastasizing cancer cells, can keep them dormant within the metastatic niche, and can impair metastatic niche development (35–38). Yet, several other types of immune cells recruited to the metastatic niche support tumor outgrowth. This includes VEGFR1 expressing macrophages that are recruited by breast cancer cells to the lung metastatic niche (39) and myeloid-derived (Gr-1^+^) cells that have been suggested to support breast cancer-derived liver metastasis growth (40). Moreover, it has been recently discovered that neutrophils are important mediators of activating dormant cancer cells in the lung metastatic niche (41). Currently, most evidence for the importance of cancer-immune cell interaction in the metastatic niche is provided through selected deletion of immune cell populations and subsequent analysis of metastasis formation (42). Thus, it will be interesting to further dissect the mechanism that allow cancer cells to rely on immune cells in the early metastatic niche. Moreover, cancer cells have developed several strategies to evade immune surveillance. Some of these mechanisms, such as reduced antigen presentation and PDL1 expression, would certainly benefit cancer cells within the metastatic niche and recent evidence suggests that these mechanisms are even enhanced during metastasis formation (43–45). Yet, it remains to be determined whether metabolic competition [an important immune evasion mechanism described in an established tumor microenvironment (46–49)] is relevant in the early metastatic niche.

Some remodeling of the extracellular matrix already occurs in the premetastatic niche, yet further changes within the metastatic niche are necessary to enable the outgrowth of cancer cells into metastases. Accordingly, it has been found that HIF1α stabilization through hypoxia and/or TGFβ upregulates the expression of the extracellular matrix modifying enzyme collagen prolyl-4-hydroxylase (P4HA) in breast cancer cells (50, 51). Consequently, high stability collagen is deposited in the lung metastatic niche supporting metastatic outgrowth. Therefore, inhibiting P4HA in cancer cells impairs breast cancer cell-derived lung metastasis formation (51, 52). Additionally, it has been recently found that P4HA is metabolically regulated by pyruvate (32), a nutrient that is particularly available in the lung (11). Consequently, inhibiting pyruvate uptake prevents collagen remodeling even in the context of HIF1α stabilization and thus is effective in impairing metastatic outgrowth of breast cancer-derived lung metastases (32). Moreover, it has been found that breast cancer and osteogenic cells form heterotypic adherens junctions in the bone metastatic niche, which enhance mTOR activity and drive early-stage bone colonization (53). Accordingly, recent data suggest that in the bone metastatic niche breast cancer cells release lactate to activate the resorption of normal collagen by osteoclasts promoting in osteolytic lesions within the bone extracellular matrix ultimately supporting metastatic growth (54). Similarly, pancreatic ductal adenocarcinoma recruit metastasis-associated macrophages to the liver metastatic niche, where they in turn activate resident stellate cells into myofibroblasts. Consequently, these myofibroblasts alter the extracellular matrix into a fibrotic environment that sustains metastatic growth (55). Finally, additional changes in cancer cell signaling within the metastatic niche can occur without the mediation of extracellular matrix. In line, it has been found that osteoblasts can secrete soluble factors that mediate dormancy of prostate cancer cells in the bone metastatic niche (56). Moreover, pericyte-like interaction of cancer cells with the endothelium has been suggested to activate yes-associated protein (YAP) signaling supporting metastatic growth (57).

Nutrient availability differs between organs and thus cancer cells need to adapt the activity of their metabolic pathway to the nutrients present in the metastatic niche (8). Consequently, the nutrient availability of the metastatic niche might enhance the organotropism of metastasizing cancer cells since certain organs provide a more permissive nutrient environment and thus result in less constrains in metabolic pathway activity (8). Additionally, nutrient enrichment results in changes in intracellular metabolite concentrations and consequently regulation of cellular programs such as signaling cascades and protein, matrix as well as DNA modifications (11, 32, 58–60). Moreover, it emerges that cancer cells in the metastatic niche cooperate with stromal cells to fuel their metabolism. Accordingly, it has been found that metastasizing ovarian cancer cells locate to omentum within the abdominal cavity to fuel on lipids released by resident adipocyte (61). During this early phase in metastatic growth ovarian cancer cells consequently can generate glycogen stores that they mobilize via interaction with cancer-associated fibroblast once nutrients get limiting (62). While multiple additional metabolic interactions have been described in an established tumor microenvironment (63) it will be important to define which of those occurs in the early metastatic niche and supports cancer cell outgrowth.

In summary, interactions in the metastatic niche are versatile and at least in part dependent on cancer cell origin, the stage of metastasis and the corresponding metastatic niche (Figure 1B). Further understanding of the dynamic changes in these interactions will be important to target them for therapy.

### Metabolic Interaction in the Primary Tumor Microenvironment vs. the Metastatic Niche

Metabolic interactions between the host stroma and cancer cells have been widely characterized in primary tumor environment (64). Thus, it is interesting to compare whether similar communications exist at the metastatic site (Table 1). In the primary tumor, cancer-associated fibroblasts (CAFs) secrete lactate, which is consumed by cancer cells in a process termed Reverse Warburg Effect (RWE) (65, 75). At the same time, hypoxic cancer cells enhance the glycolytic activity of CAFs, contributing indirectly to this process (76). Moreover, it has been reported that CAFs and cancer-associated adipocytes (CAAs) boost lipid metabolism in breast cancer and melanoma cells by inducing the expression of the fatty acid transporter FATP1 (66, 68). However, if these mechanisms are also employed during metastatic progression is still not clear. It has been shown that in the premetastatic niche fibroblasts increase glucose availability to resident lung cells (34), suggesting that a metabolic crosstalk between fibroblasts and cancer cells might be a relevant regulator of metastatic growth. CAAs when co-cultured with ovarian cancer cells sustain lipid metabolism via the expression of CD36 (67). Similarly, CAAs also provide fatty acids as a source of energy for β-oxidation in omental metastases (61, 69). This interaction can explain the observation that intra-abdominal tumors show a metastatic preference for the omentum, which is mainly composed of adipocytes. Further investigation is needed to address whether CAAs also alter lipid metabolism in other metastatic sites.

**Table 1:** Cellular interaction within the primary tumor and metastases environment.

**Cell type**	**Primary tumor**	**Metastasis**	**Effects on cancer cells**
Fibroblasts	Provide lactate by undergoing RWE (65) Induce expression of FA transporter in breast cancer (66)	Provide increased glucose availability by decreasing their own glucose utilization (34)	PRO-TUMOR
Adipocytes	Induce CD36 expression in ovarian cancer (67) and FATP1 in melanoma (68)	Undergo lipolysis to fuel β-oxidation in omentum metastasis (61, 69)	
Tregs	Survival favored in high lactate environment (70)	Inhibit anti-tumor immune cells	
M2 macrophages	M2 polarization favored by lactate (70)	Actively recruited in the metastatic and pre-metastatic niche (71, 72)	
MDSCs	Proliferation induced by lactate (70)	Major regulators of pre-metastatic niche formation (73)	
NK cells	Mediate cytotoxic killing of cancer cells, but infiltration and activation is inhibited by lactate (70) and adenosine in the tumor environment (47)	Impaired by upregulation of inhibitory receptors on cancer cells (44)	ANTI-TUMOR
T-cells	Mediate cytotoxic killing of cancer cells, but infiltration and activation is inhibited by depletion of glucose, tryptophan and arginine or accumulation of glutamate (47) and lactate in the tumor environment (70)	Reduced density (74) and downregulated HLA1 expression (45)	

*FA refers to fatty acid, FATP1 refers to Fatty Acid Transport Protein 1, MDSC refers to myeloid-derived suppressor cell, NK refers to natural killer cell, RWE refers to reverse Warburg effect, Tregs refers to regulatory T-cells*.

In addition, several studies have addressed the metabolic crosstalk between cancer cells and immune cells in the primary tumor environment. T-cells display reduced functionality owing to a deprivation of essential metabolites such as glucose, arginine and tryptophan or the accumulation of unwanted by products such as lactate (48). This also leads to the expansion of pro-tumor cell types such as myeloid-derived suppressor cells (MDSCs) and regulatory T-cells (Tregs) (47). Lactate accumulation also has an inhibitory effect on natural killer (NK) cell activity while concomitantly promoting expansion of the pro-tumor MDSCs, M2 polarized macrophages and Treg populations (70). However, it remains largely elusive whether the same alterations occur in the (pre)metastatic niche. Interestingly, the metastases environment displays a reduced number of tumor-infiltrating lymphocytes as compared to primary tumor environment, and can be associated with reduced overall survival (43, 74, 77). Yet, the mechanisms that give rise to these differences are not fully understood. Macrophages and MDSCs are actively recruited at the primary tumor, premetastatic, and metastatic niches and act as key drivers of the metastatic seeding and progression via a plethora of different mechanisms. Accordingly, it has been reported that their depletion leads to inhibition of metastatic progression (71–73). However, further studies are required to address the specific differences between macrophages that infiltrate the primary tumor and metastases.

Taken together, it will be interesting to investigate the contribution of metabolic factors in driving the above-mentioned variations in the immune cell population in the metastatic niche.

### Therapeutic Strategies Targeting Metastasis Formation

Mortality after progression of cancers to a metastatic disease is primarily caused by the lack of effective treatments. Many secondary tumors are resistant to chemotherapy and in some cases, chemotherapy might even promote metastasis formation (78, 79). Thus, there is a rising need for novel approaches to target metastasis formation. Recent findings suggest that targeting nutrient metabolism might be a promising strategy to overcome the lack of treatments against metastatic progression (8, 80). Accordingly, a fructose-restricted diet suppressed liver metastases more efficiently than the first-line treatment with the chemotherapeutic agents 5-Fluorouracil and oxaliplatin (59). Moreover, several studies have reported a strong negative correlation between infiltration of T-cells and NK cells and metastatic growth (37, 43, 81, 82). Checkpoint blockade therapy to activate the infiltrating T cells has shown promising results, particularly in advanced metastatic melanoma (83, 84). Increasing the repertoire of tumor infiltrating T-cells and NK cells by adoptive transfer of *ex vivo* expanded cells shows promising effects particularly against metastatic melanoma, pulmonary metastases of Ewing sarcoma as well as anaplastic thyroid cancer, and systemic metastases of glioblastoma (85, 86). These new strategies are emerging as promising therapeutic approaches that consider not only the molecular phenotype of metastatic tumors but also the environment of the metastatic niche. Further studies and clinical trials are needed to develop effective therapies against metastasis formation.

## Concluding Remarks

(Pre)metastatic niche formation is an important step in the metastatic cascade. Targeting the interactions that build the (pre)metastatic niche has the potential to prevent and eradicate metastases before they manifest. However, multiple questions remain and need to be investigated to translate our current knowledge of (pre)metastatic niche formation toward clinical impact. For instance, which primary tumors secrete factors that support metastatic niche formation. This is important to stratify patients, since it has been found that the presence of certain primary tumors can inhibit metastasis formation through a process called concomitant immunity (7). Moreover, it will be important to study the dynamics of (pre)metastatic niche formation to determine which interaction should be targeted in cancer patients of different disease stage. To succeed in this, it is important to develop biomarkers and tools that allow to assess the extent of (pre)metastatic niche formation in patients. Moreover, organ-specific treatment of metastases and their niche is required, especially when targeting metabolic rewiring which is a direct function of the available nutrients (8, 32, 58, 60, 87, 88). Finally, clinical trials that assess treatments for metastasis prevention are required to bring the bench to the bedside.

## Author Contributions

GD, SP, and S-MF wrote and edited the manuscript and made a substantial direct and intellectual contribution to the work. All authors approved it for publication.

### Conflict of Interest Statement

S-MF has received research funding from Bayer AG and Merck. The remaining authors declare that the research was conducted in the absence of any commercial or financial relationships that could be construed as a potential conflict of interest.
